# The Effect of Phototherapy on Systemic Inflammation Measured with Serum Vitamin D-Binding Protein and hsCRP in Patients with Inflammatory Skin Disease

**DOI:** 10.3390/ijms25168632

**Published:** 2024-08-08

**Authors:** Andrea Elmelid, Maria Siekkeri Vandikas, Martin Gillstedt, Mikael Alsterholm, Amra Osmancevic

**Affiliations:** 1 Department of Dermatology and Venereology, Institute of Clinical Sciences, Sahlgrenska Academy, University of Gothenburg, 413 45 Gothenburg, Sweden; maria.s.vandikas@gu.se (M.S.V.); martin.gillstedt@vgregion.se (M.G.); mikael.alsterholm@vgregion.se (M.A.); 2 Center for Clinical Research Dalarna, Uppsala University, 791 82 Falun, Sweden; 3 Dermatology and Venereology Unit, Karolinska University Hospital, 171 76 Stockholm, Sweden; 4 Department of Dermatology and Venereology, Sahlgrenska University Hospital, Region Västra Götaland, 413 45 Gothenburg, Sweden; 5 Division of Dermatology and Venereology, Department of Medicine Solna, Karolinska Institutet, 171 76 Stockholm, Sweden

**Keywords:** vitamin D, vitamin D binding protein (gc-globulin), immunomodulation, free 25-hydroxy vitamin D, psoriasis, atopic dermatitis, phototherapy, ultraviolet B therapy, inflammation

## Abstract

Vitamin D plays a role in inflammatory skin disease, but the exact mechanisms and the clinical significance remain unclear. According to the free hormone hypothesis, it is the free concentration of 25-hydroxy vitamin D (25(OH)D) that is biologically active. Vitamin D-binding protein (DBP) acts as the major transporter of vitamin D in the circulation, and DBP concentration defines the free 25(OH)D levels. DBP levels are elevated in various inflammatory conditions, including psoriasis. Narrowband-ultraviolet B (NB-UVB) is the most widely used phototherapy and is an established first-line treatment for psoriasis and atopic dermatitis (AD), often used before proceeding to systemic treatment. The aim of this study was to investigate the influence of NB-UVB phototherapy on DBP and high-sensitivity C-reactive protein (hsCRP) levels, as markers of systemic inflammation, in inflammatory skin disease. Thirty adults (psoriasis (*n* = 20) and AD (*n* = 10)) were treated with NB-UVB. Serum DBP, hsCRP, total and free 25(OH)D, and 1,25-dihydroxy vitamin D (1,25(OH)_2_D) were measured before and after NB-UVB. Disease severity was assessed with Psoriasis Area and Severity Index (PASI), SCORing Atopic Dermatitis (SCORAD), and Visual Analogue Scale (VAS). DBP decreased in psoriasis patients and varied with no clear trend in AD patients. HsCRP decreased in both groups, but this did not reach statistical significance. PASI, SCORAD, and VAS improved, and vitamin D levels increased after NB-UVB. Sub-analysis indicated a better response to NB-UVB for patients with vitamin D deficiency and insufficiency compared to vitamin D-sufficient patients. The decrease in DBP after NB-UVB in psoriasis patients suggests a potential systemic anti-inflammatory effect of phototherapy. Measurement of vitamin D levels may potentially serve as a tool to identify patients who would derive the greatest benefit from NB-UVB phototherapy.

## 1. Introduction

Vitamin D plays a complex and multifaceted role in the context of skin health [1]. Its involvement in the common inflammatory skin diseases such as psoriasis and atopic dermatitis (AD) has been subject to extensive research, yet the significance of vitamin D in the pathogenesis of these two diseases remains unclear.

Vitamin D influences immune cell activity, particularly T cells, which are central to psoriasis lesion development [2]. Additionally, vitamin D regulates the growth and differentiation of keratinocytes which are key processes in psoriasis pathogenesis. Topically applied synthetic vitamin D analogues prevent abnormal keratinocyte proliferation in psoriasis, while the use of oral vitamin D has shown inconsistent treatment results [3,4,5]. 

The role of vitamin D in AD pathogenesis is debated, as studies have generated conflicting data. A disrupted skin barrier is a characteristic feature of AD. A function for vitamin D in the maintenance and restoration of the skin barrier is suggested by its influence on keratinocyte differentiation, tight junctions, filaggrin synthesis, immune modulation, wound healing, and anti-inflammatory effects [1,6]. Populations living at latitudes with lower sun exposure and therefore possibly lower vitamin D levels have increased AD prevalence [7]. A meta-analysis found that patients with AD have lower serum 25-hydroxy vitamin D (25(OH)D) levels compared to controls across all ages (standardized mean difference = −2.03 ng/mL; 95% CI = −2.98 to −1.08), but the difference was more pronounced in pediatric patients (standardized mean difference = −3.03 ng/mL, 95% CI = −4.76 to −1.29) [8]. In contrast, one study found no association between 25(OH)D levels and AD, while another found no link between the prevalence and incidence of AD and 25(OH)D levels [9,10]. Vitamin D supplementation reduces AD symptoms according to a meta-analysis including four randomized controlled trials (RCTs). The study showed significant decreases in both the SCORing Atopic Dermatitis (SCORAD) index and the Eczema Area and Severity Index (EASI) (mean difference = −5.85, 95% CI = −7.66 to −4.05) [8].

The multi-etiology of vitamin D deficiency among patients with AD and psoriasis contributes to the diversity in study findings. Confounding factors such as skin type, geographic location, diet, age, comorbidities, and medications can significantly influence vitamin D levels. Additionally, variations in sun exposure and genetic predispositions further complicate the understanding of vitamin D deficiency in these populations [11]. 

Ultraviolet light B (UVB) exposure, leading to vitamin D synthesis in the skin, raises serum 25(OH)D levels, and is an effective treatment for both psoriasis and AD [12,13,14]. The reason why patients with AD and psoriasis have lower vitamin D levels than healthy individuals is not fully understood. One possible explanation is that the thickened and inflamed skin resulting from these conditions impairs local vitamin D production in the skin. This theory is supported by findings from a study that compared the increase in serum 25(OH)D levels after phototherapy among patients with AD, psoriasis, and healthy controls. The study found that healthy individuals had the highest increase in 25(OH)D levels, followed by AD patients, with the lowest increase observed in patients with psoriasis [13].

Narrow-band UVB (NB-UVB) phototherapy is well tolerated and has few adverse effects as long as safety protocols and precautions are implemented. Possible side effects of NB-UVB include sunburn-like erythema, reactivation of herpes infections, and keratitis or conjunctivitis if eye protection is inadequate. Phototoxic or photoallergic reactions are rare since they usually have an action spectrum within the UVA range. The risk of photocarcinogenesis with NB-UVB seems to be negligible [15], but higher rates of actinic keratoses have been reported [16,17].

The beneficial effects of UV radiation (UVR) on body homeostasis extend beyond vitamin D synthesis and depend on chromophores and the depth of UVR penetration [18]. While previous research has focused on the total 25(OH)D levels, recent evidence suggests that it might be more adequate to measure the free 25(OH)D levels in the assessment of vitamin D status. Total 25(OH)D is the sum of the 25(OH)D tightly bound to vitamin D binding protein (DBP) (~85%), the fraction loosely bound to albumin (~15%), and a small amount (~0.03%) circulating freely. According to the free hormone hypothesis, it is the free fraction of a hormone that is biologically active. For other steroid hormones, it is the free fraction that is measured in clinical practice [19]. DBP null mice and humans with DBP deficiency have a normal phenotype but extremely low total 25(OH)D and 1,25-dihydroxy vitamin D (1,25(OH)_2_D), strongly supporting the free hormone hypothesis for vitamin D [20,21].

There is limited knowledge about the significance of DBP in the context of inflammatory skin diseases. In a recent study, psoriasis patients had higher DBP serum levels compared to healthy controls [22]. In another study, higher serum levels of DBP were observed in individuals with psoriasis reporting arthropathy compared to those who did not [23]. Moreover, elevated serum DBP levels have been found in cardiovascular disease and inflammatory bowel disease, which are known comorbidities of psoriasis and AD. DBP could potentially serve as an inflammation marker in these conditions [24,25]. To the best of our knowledge, there are no data on DBP levels in AD patients in the literature.

In this interventional cohort study, it was hypothesized that treatment with NB-UVB would decrease DBP levels in patients with psoriasis and AD since phototherapy reduces skin inflammation [13,26,27]. The secondary hypothesis was that there are positive associations among serum levels of DBP, high-sensitivity C-reactive protein (hsCRP), and disease severity.

## 2. Results

### 2.1. Demographics and Baseline Data

Thirty patients with inflammatory skin disease (plaque-type psoriasis *n* = 20, AD *n* = 10) participated in the study, with 23 (psoriasis *n* = 15, AD *n* = 8) completing the entire protocol. The mean age was 39 ± 15 (standard deviation (SD)) years. The psoriasis patients were older (mean age 44 ± 16 years) than the AD patients (mean age 29 ± 7 years). A majority of the patients (*n* = 27 (90%)) were enrolled in the study in winter (October–March).

There were eleven patients (37%) with sufficient vitamin D levels (25(OH)D ≥ 75 nmol/L) at baseline, while 19 (63%) patients had either deficiency (25(OH)D < 50 nmol/L) (*n* = 7 (23%)) or insufficiency (25(OH)D = 50–74 nmol/L) (*n* = 12 (40%)) [28].

Most patients were of normal weight and had normal blood pressure. Demographic and baseline data including concomitant medication and potential confounders for vitamin D status are presented in Table 1. 

### 2.2. Effects of NB-UVB Phototherapy on Serum DBP, hsCRP, Vitamin D, and Disease Severity

Serum levels of DBP decreased in patients with psoriasis (*p* = 0.031) and varied with no clear trend in patients with AD. HsCRP decreased in both groups, but this did not reach statistical significance (Table 2).

Total 25(OH)D, free 25(OH)D, and 1,25(OH)_2_D increased after phototherapy (visit 3, Week 10–12) (*p* < 0.0001, *p* < 0.0001, and *p* = 0.009, respectively). The intact parathyroid hormone (iPTH) decreased (*p* = 0.020). Calcium and albumin remained unaltered.

**Figure 1:**
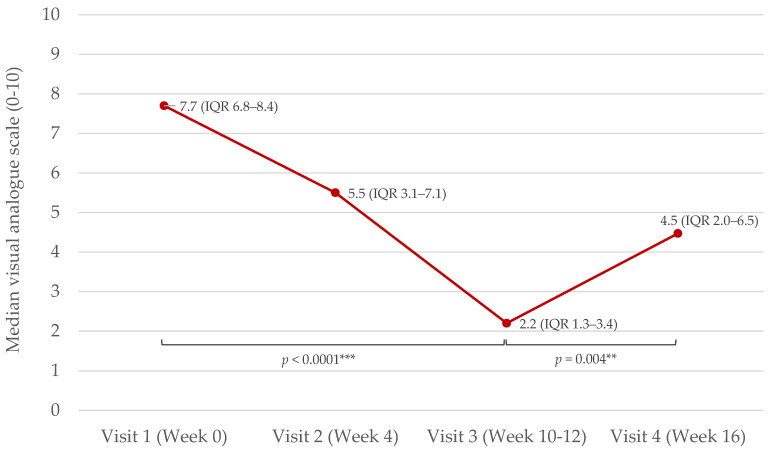
Effect of narrow-band ultraviolet light B (NB-UVB) on severity of inflammatory skin disease (psoriasis and atopic dermatitis (AD)) measured with visual analogue scale (VAS). NB-UVB was started after VAS-assessment at visit 1 and stopped at visit 3. Visit 4 was the posttreatment follow-up. Median values (inter-quartile range (IQR)) are presented. Spearman’s correlation test stratifying with respect to the patient was used. The significance levels were defined as *p* < 0.01 (**) and *p* < 0.001 (***).

Disease severity (measured with visual analogue scale (VAS)) decreased from visit 1 (week 0) to visit 3 (at week 10–12 when NB-UVB was ended) (*p* < 0.0001). VAS increased from visit 3 to visit 4 (week 16, 4–6 weeks after cessation of phototherapy) (*p* = 0.004) (Figure 1).

PASI (Psoriasis Area and Severity Index) and objective SCORAD decreased significantly as previously reported separately for psoriasis and AD patients. Median PASI decreased from 8.4 (7.6–12.2) to 2.6 (1.2–3.2), and median objective SCORAD decreased from 37.1 (28.9–38.4) to 19.8 (17.0–22.4) [29,30].

#### 2.2.1. Effect of NB-UVB Phototherapy on Disease Severity, DBP, hsCRP, and Vitamin D after Stratification by Baseline Vitamin D Levels

**Figure 2:**
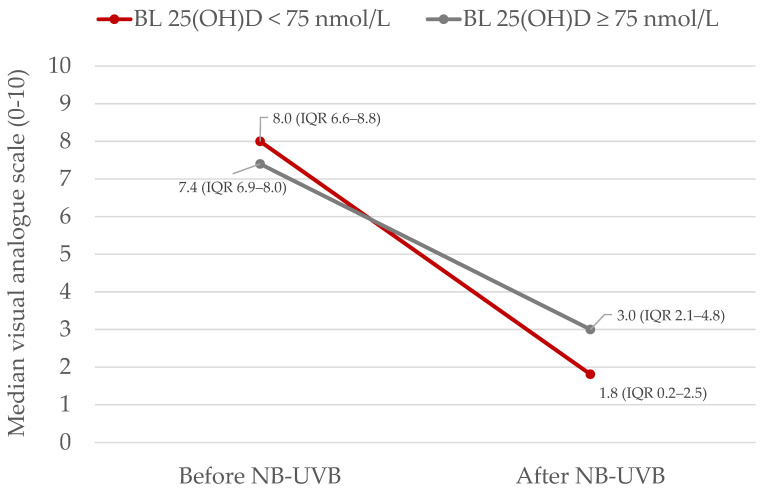
Effect of narrow-band ultraviolet light B (NB-UVB) on severity of inflammatory skin disease measured with visual analogue scale (VAS) stratified by baseline (visit 1 = week 0) vitamin D levels. The VAS score decrease was greater (*p* = 0.038) in the vitamin D deficient/insufficient group (red, *n* = 14) compared to the vitamin D sufficient group (grey, *n* = 9). Median values (inter-quartile range (IQR)) are presented.

Patients with vitamin D deficiency or insufficiency (25(OH)D < 75 nmol/L), before phototherapy (visit 1 = week 0), had a significantly better response to treatment (measured with delta VAS) compared to patients with vitamin D sufficiency (25(OH)D ≥ 75 nmol/L), (*p* = 0.038) (Figure 2).

Baseline vitamin D levels did not affect the magnitude of PASI score change during phototherapy in psoriasis patients.

The median objective SCORAD change was −23.7 (IQR (−24.5, −18.9)), (*n* = 3), in the vitamin D deficient/insufficient AD patients and −16.5 (IQR (−17.4, −4.4)), (*n* = 5), in the vitamin D sufficient AD patients (*p* = 0.46).

DBP decreased significantly in the vitamin D deficient/insufficient group (*p* = 0.028) but not in the vitamin D sufficient group. The mean DBP decrease was −6.8 ± 9.7 in the deficient/insufficient group, compared to 2.7 ± 17.4 in the vitamin D sufficient group. However, there was no significant difference between the groups regarding DBP change after phototherapy (*p* = 0.18).

There was no difference in hsCRP change after phototherapy between vitamin D deficient/insufficient versus vitamin D sufficient patients (*p* = 0.93).

The 25(OH)D levels at baseline did not affect the absolute increase in any of the measured vitamin D metabolites (total and free 25(OH)D, 1,25(OH)_2_D and percentage of free 25(OH)D).

#### 2.2.2. Effect of NB-UVB Phototherapy on Vitamin D, DBP, and hsCRP after Stratification by Baseline Body Mass Index (BMI)

Neither obesity (BMI > 30 kg/m^2^) nor being overweight (BMI > 25 kg/m^2^) affected the increase in vitamin D levels (total and free 25(OH)D, 1,25(OH)_2_D, and percentage of free 25(OH)D) after phototherapy. DBP and hsCRP changes were unaffected by the baseline BMI.

#### 2.2.3. Baseline DBP, hsCRP, and Disease Severity, and Effect of NB-UVB Phototherapy on DBP and hsCRP in Psoriasis Patients after Stratification by Self-Reported Arthropathy

Patients with psoriasis reporting arthropathy had significantly higher baseline DBP levels compared to those not reporting arthropathy (*p* = 0.044). There were no differences between the groups regarding hsCRP levels or disease severity (PASI and VAS) at baseline. There was a tendency towards larger DBP decrease after NB-UVB in the group reporting arthropathy compared to the group without arthropathy (*p* = 0.066). Arthropathy did not affect the changes in the hsCRP levels.

### 2.3. Baseline Correlations

All measured vitamin D metabolites were positively correlated to DBP at baseline (visit 1 = week 0). DBP and hsCRP were not correlated when testing the entire group of inflammatory skin disease patients. However, in a sub-analysis, there was a trend towards a positive association between DBP and hsCRP in AD patients (*p* = 0.054). There were no correlations between disease severity and hsCRP or DBP, respectively (Table 3).

HsCRP and BMI were positively correlated (*p* = 0.012). BMI and percentage of the free 25(OH)D were negatively associated (*p* = 0.0005).

## 3. Discussion

This study presents the effects of NB-UVB phototherapy on serum levels of DBP, hsCRP, vitamin D, and disease severity in a cohort of patients with psoriasis and AD.

DBP decreased after NB-UVB phototherapy in psoriasis patients, indicating that phototherapy might have a systemic anti-inflammatory effect in these patients. This is in contrast to a previous study on psoriasis patients with similar disease severity, where the DBP levels remained unaltered by phototherapy [22]. The reason for the contradictory results regarding the impact of phototherapy on DBP levels is not apparent. In the previous study, pretreatment levels of 25(OH)D were notably higher compared to our data (mean 90 ± 35 nmol/L vs. 67.7 ± 21.0 nmol/L), which might influence the results. This explanation is further supported by our finding that, after stratifying the cohort (including both psoriasis and AD patients) by the baseline vitamin D levels, DBP decreased significantly in the vitamin D deficient/insufficient group but not in the vitamin D sufficient group. Additionally, DBP levels were higher in the previous cohort, and no correlation was observed between DBP and total 25(OH)D levels. In contrast, our study found positive correlations between DBP and all measured vitamin D metabolites, consistent with expectations. The conflicting results might also be attributed to differences in phototherapy regimens, as the previous study used both broadband UVB and NB-UVB.

HsCRP, which is a marker of residual inflammation [31], decreased in this cohort of inflammatory skin disease patients, but the decrease did not reach statistical significance. CRP levels have been shown to decrease after NB-UVB in patients with psoriasis [32]. A previous trial investigated the effects of NB-UVB phototherapy on CRP, and several additional cardiovascular risk markers. There was a decrease in CRP levels, while the other cardiovascular risk markers were unaltered by phototherapy [33]. To evaluate the systemic effects of NB-UVB and other treatments, larger and randomized studies are needed, focusing on markers for systemic inflammation, cardiovascular risk, vitamin D, and related molecules. Moreover, UVR has a broader impact on health involving mechanisms beyond UVB-induced vitamin D production. The skin acts as a neuroendocrine organ, releasing hormones, neurotransmitters, and cytokines in response to UVR, resulting in various local or central responses depending on wavelength. These responses affect body homeostasis by modulating immune functions, activating neuroendocrine pathways like the hypothalamic–pituitary–adrenal (HPA) axis, the hypothalamic–pituitary (HP) axis, and potentially influencing mood and behavior. Understanding the detailed mechanisms of UVR’s effects on the neuro-immuno-endocrine system can lead to novel therapeutic approaches extending beyond skin conditions to systemic treatments for autoimmune diseases, neurodegenerative disorders, and mood disorders [18].

As reported earlier, NB-UVB phototherapy led to significant clinical improvements [29,30] but also a notable resurgence in disease severity mere weeks after treatment cessation. The durability of the phototherapy effect needs to be critically appraised in the era of rapidly improving systemic pharmacotherapy for psoriasis and AD.

This study confirms significant increases in total 25(OH)D, free 25(OH)D, and 1,25(OH)_2_D serum levels after phototherapy, indicating its positive effects on systemic vitamin D levels, as previously reported [12,34]. Patients with lower vitamin D levels at the baseline demonstrated a better treatment response, reported as a greater decrease in VAS scores, suggesting the importance of vitamin D status in therapeutic outcomes and the potential of utilizing vitamin D levels as a predictor for phototherapy effectiveness. Moreover, optimal vitamin D serum levels may protect psoriasis patients from trigger-induced flares. A retrospective study on psoriasis exacerbation following COVID-19 vaccination found higher rates of exacerbation in patients with low vitamin D levels. In contrast, lower exacerbation rates were observed during the summer, a season characterized by extensive sun exposure [35].

Conflicting results in previous studies regarding phototherapy efficacy might be due to the lack of pretreatment stratification for vitamin D levels. Patients with sufficient 25(OH)D levels have likely reached a plateau in the sigmoid response curve for vitamin D’s biological effects and will not benefit from further increases in vitamin D levels [36].

Furthermore, future research should investigate the effects of UVB phototherapy in the skin, particularly focusing on CYP11A1, a crucial member of the cytochrome P450 family. The recent discovery of alternative pathways for vitamin D3 activation by CYP11A1, producing at least 18 biologically active hydroxyderivatives, has significantly advanced vitamin D research. These derivatives act through various nuclear receptors not limited to VDR, resulting in different receptor-specific biological effects. Additionally, vitamin D3 is not the only photoproduct of UVB from 7-dehydrocholesterol (7-DHC); lumisterol and tachysterol are also activated to biologically active forms with both overlapping and distinct effects from 1,25(OH)_2_D [37]. CYP11A1 expression is decreased in patients with AD and psoriasis and may play a role in the pathogenesis of inflammatory skin diseases [38,39]. UVB exposure stimulates CYP11A1 and cortisol production in the skin, which might contribute to the improvement in skin condition for these patients. CYP11A1 plays a vital role in the skin by initiating local steroidogenesis and metabolizing vitamin D, lumisterol, and 7-DHC. The products of these pathways regulate the skin’s protective barrier and immune response through interactions with various receptors [40]. The hydroxymetabolites of vitamin D3, 20(OH)D3 and 20,23(OH)_2_D3, produced by CYP11A1, exhibit anti-inflammatory and antioxidant properties, similar to those observed in the traditional active forms of vitamin D3 [41].

DBP serum levels have been positively correlated to hsCRP serum levels in healthy individuals, but this could not be confirmed in this study, possibly due to the small sample size [42]. However, sub-analysis suggested a potential positive association between DBP and hsCRP in AD patients. Additionally, there was a trend towards a more significant reduction in DBP levels after phototherapy in patients with arthropathy compared to those without, further supporting this hypothesis. This finding aligns with a previous study, which reported higher serum DBP levels in psoriasis patients with arthropathy compared to those without. Furthermore, a significant reduction in DBP levels was observed in the arthropathy group following etanercept treatment [43]. However, the evaluation of DBP as an inflammatory marker in psoriasis and AD needs to be studied in larger cohorts, ideally in the setting of an interventional study design studying the effects of different treatment regimes.

Interestingly, there was a negative correlation between the percentage of free 25(OH)D and BMI, suggesting that lower BMI is associated with a higher proportion of free (and bioactive) vitamin D and vice versa. This contrasts with a previous report on healthy blood donors from the same geographic area and with a similar mean age as the present cohort, where BMI and the percentage of free 25(OH)D were positively associated [42]. Our finding is supported by previous research demonstrating that DBP binds to fatty acids; and that unsaturated fatty acids impair DBP’s binding to 1,25(OH)_2_D and 25(OH)D [44,45]. HsCRP levels were positively associated to BMI as expected and previously demonstrated [46]. Obesity is a systemic inflammatory condition associated with decreased serum concentrations of total 25(OH)D, likely attributable to its lipophilic nature and sequestration within adipose tissue [47,48]. Moreover, obesity and being overweight constitute independent risk factors and aggravators of psoriasis [49,50]. Recent evidence points to a synergistic effect of obesity and low vitamin D levels in relation to psoriasis. A prospective cohort study showed that individuals with 25(OH)D deficiency (<25 nmol/L) had a 20% higher risk of developing psoriasis. The highest risk was observed in the obese group (BMI > 30 kg/m^2^), which had a 30% increased risk compared to those with 25(OH)D levels above 50 nmol/L [51]. Additionally, a large cross-sectional cohort study discovered a super additive effect where high BMI combined with low 25(OH)D level significantly increased the risk of active psoriasis [52]. Our findings suggest that elevated BMI not only influences the total vitamin D levels but also negatively alters the bioactive levels of vitamin D. This might be a key to the understanding of underlying pathways of the causality between obesity and psoriasis. In contrast to previous studies showing decreased response to oral vitamin D substitution in overweight and obese individuals, compared to individuals of normal weight, we observed that BMI did not affect the skin’s ability to produce vitamin D [53]. A potential clinical implication of these insights is individualized treatment with vitamin D substitution or NB-UVB phototherapy for patients with inflammatory skin disease and concomitant obesity.

The main limitation of this study is the small sample size. The results of inferential baseline statistics on a cohort of this size have to be interpreted with caution. With this in mind, we found several significant correlations that may be of importance to understand the interplay of UVB radiation and vitamin D metabolism in humans. The small size of the study is balanced by its interventional design, where all patients received the same type of phototherapy (NB-UVB). This approach establishes temporality, offering a notable advantage. The method utilized for measuring 25(OH)D levels (ECLIA) is not considered the gold standard, such as liquid chromatography–tandem mass spectrometry (LC-MS/MS). However, it is a validated method widely used in the assessment of vitamin D status [54]. To evaluate the duration of the NB-UVB treatment effect, it would have been optimal to measure not only VAS but also PASI and SCORAD at the 16-week follow-up. However, due to practical constraints and to minimize additional clinic visits for patients, this was not performed.

## 4. Materials and Methods

### 4.1. Study Design, Setting, and Patients

Adult (≥18 years) patients with inflammatory skin disease for whom phototherapy was indicated were asked to participate. In total, 30 patients were included (20 with mild to severe chronic plaque psoriasis, and 10 with moderate to severe AD). The subjects were described in detail earlier [29,30].

The exclusion criteria were as follows: pregnancy or lactation, severe chronic or systemic disease, treatment with systemic corticosteroids, other immunosuppressive/anti-inflammatory drugs, or antibiotics. Sunbed use or sun holidays were not allowed four weeks before inclusion. Vitamin D supplements were not permitted during the study period or in the four weeks prior to inclusion.

A comprehensive questionnaire was used to collect data about medical history, medication use, dietary supplements, sun exposure habits, and other lifestyle variables potentially influencing vitamin D status, DBP levels, and inflammation.

The patients received NB-UVB phototherapy according to standard procedure for 10–12 weeks as previously described [29,30]. Patients recruited from October to March were defined as winter recruited, based on the UV index (<3) in Gothenburg. Patients recruited from April to September, when UV index can reach >3 and vitamin D production in the skin is possible, were classified as recruited in summer.

Patients were stratified by baseline vitamin D levels into two groups, those with serum 25(OH)D levels ≥75 nmol/L (sufficiency) and those with levels <75 nmol/L (including both deficient and insufficient patients), following reference values outlined by the Endocrine Society. Deficiency was defined as 25(OH)D < 50 nmol/L [28].

### 4.2. Laboratory Analyses

Biochemical analyses were performed at the Department of Clinical Chemistry, Sahlgrenska University Hospital, Gothenburg.

Serum levels of DBP, hsCRP, total and free 25(OH)D, 1,25(OH)_2_D, iPTH, calcium, and albumin were measured before and after NB-UVB treatment.

DBP was analyzed with a monoclonal enzyme-linked immunoassay (ELISA) (R&D systems, Minneapolis, MN, USA).

Serum levels of hsCRP, calcium, and albumin were analyzed with standardized laboratory techniques on a Cobas 8000 Roche instrument (Roche Diagnostics Scandinavia AB, Tokyo, Japan).

To analyze the total 25(OH)D [25(OH)D2 and 25(OH)D3] serum levels, an Electrochemiluminescence immunoassay (ECLIA) (Elecsys Vitamin D Total II assay) on a Cobas 8000 Roche instrument (Roche Diagnostics Scandinavia AB, Tokyo, Japan) was used.

A two-step immunosorbent assay (ELISA) (Future Diagnostics B.V., Wijchen, The Netherlands) was used to measure the free 25(OH)D serum levels.

An automated chemiluminescence immunoassay (CLIA) was used to analyze serum 1,25(OH)_2_D with an IDS-iSYS instrument (IDS, Boldon, UK). 

Serum iPTH was analyzed with ECLIA with an Elecsys PTH STAT (Roche Diagnostics Scandinavia AB, Tokyo, Japan). 

### 4.3. Assessment of Disease Severity

Investigator-reported outcome measures for disease severity were the PASI in patients with psoriasis and the objective SCORAD in patients with AD.

Patient-reported disease severity was captured using the VAS, where patients indicated the intensity of their symptoms on a ten-centimeter line, with 0 representing no complaints and 10 representing the most severe complaints. VAS has been previously utilized to assess psoriasis severity and has demonstrated a strong correlation with PASI and the Dermatology Life Quality Index (DLQI) [55].

### 4.4. Statistics

Data were analyzed using R version 3.5.3 (The R Foundation for Statistical Computing, Vienna, Austria). Simple descriptive statistics were applied. Spearman’s correlation test was used to test univariate correlations. Wilcoxon’s rank-sum test was used for two sample tests. Spearman’s correlation test, stratifying with respect to the patient, was used when testing for changes in VAS over time. Wilcoxon’s signed-rank test was used to test differences in biochemical data before and after NB-UVB. All tests were two-sided, and *p* < 0.05 was considered statistically significant.

### 4.5. Ethical Considerations

The study was approved by the Ethics Committee at the University of Gothenburg on 22 May 2012 (approval number: 089-12). Declarations of Helsinki protocols were followed. Written informed consent was obtained from all patients.

## 5. Conclusions

Our findings suggest that NB-UVB phototherapy might exert a systemic anti-inflammatory effect by reducing DBP levels in psoriasis patients. Individuals with vitamin D deficiency or insufficiency seem to benefit more from phototherapy, highlighting the role of vitamin D in ameliorating inflammatory skin diseases. Furthermore, evaluating pretreatment vitamin D status could enable more precise, patient-specific therapeutic decisions.

We observed a negative association between the percentage of free 25(OH)D levels and BMI at baseline, suggesting that obesity may negatively affect the bioactive levels of vitamin D. However, unlike oral supplementation where higher BMI reduces the ability to increase serum vitamin D levels, BMI does not affect the skin’s ability to produce vitamin D. Our results indicate complex interrelationships among vitamin D metabolites, DBP, inflammation, and BMI in patients with psoriasis and AD. Further investigation is warranted to elucidate these associations and their clinical implications.

## Figures and Tables

**Table 1:** Demographic and baseline data including potential confounders for vitamin D status in thirty patients with inflammatory skin disease. Demographic data for each diagnosis (atopic dermatitis and psoriasis) have been previously published separately [29,30].

	Mean	Min–Max	*n*
Age (years)	39	20–70	30
Age women (years)	34	20–65	14
Age men (years)	43	21–70	16
Weight (kg)	74	49–111	30
Height (cm)	172	154–189	30
BMI (kg/m^2^)	25.1	19.3–33.5	30
Systolic blood pressure (mmHg)	122	90–165	30
Diastolic blood pressure (mmHg)	77	60–95	30
Disease duration of psoriasis or atopic dermatitis (years)	21	1–46	30
	*n* (%)		
Current smokers	10 (33%)		
Omega 3 use	2 (7%)		
Obesity (BMI > 30 kg/m^2^)	5 (17%)		
Skin type			
II	11 (37%)		
III	18 (60%)		
IV	1 (3%)		
Concomitant medication			
Antilipidemic use	1 (3%)		
Antihypertensive use	3 (10%)		
Antidiabetic use	1 (3%)		
Antidepressant use	2 (7%)		
Painkiller use	2 (7%)		
Thyroid hormone use	2 (7%)		
Hormonal contraception	1 (3%)		
Total 25(OH)D < 75 nmol/L (deficiency/insufficiency) Total 25(OH)D ≥ 75 nmol/L (sufficiency)	19 (63%) 11 (37%)		

**Table 2:** Serum levels of vitamin D binding protein (DBP) and high-sensitivity C-reactive protein (hsCRP) in patients with psoriasis and atopic dermatitis (AD) before and after narrow-band ultraviolet light B (NB-UVB) phototherapy. Treatment ended at visit 3 (week 10–12). Due to seven dropouts, posttreatment data are available for *n* = 23 patients. Mean values ± standard deviation (SD) and *p*-values for the trend over time are presented. * *p*-value < 0.05 using Wilcoxon’s signed rank test, for comparison of changes over time within each group.

	Before NB-UVB (Visit 1 = Week 0)	After NB-UVB (Visit 3 = Week 10–12)	*p*-Value
Psoriasis patients	*n* = 20	*n* = 15	
DBP (μg/mL)	218.0 ± 38.9	201.2 ± 16.7	0.031 *
hsCRP (mg/L)	2.6 ± 2.7	2.0 ± 1.9	0.31
AD patients	*n* = 10	*n* = 8	
DBP (μg/mL)	247.6 ± 39.8	248.6 ± 47.0	0.58
hsCRP (mg/L)	3.3 ± 3.4	2.5 ± 1.9	0.68
All patients	*n* = 30	*n* = 23	
DBP (μg/mL)	227.8 ± 41.0	201.2 ± 16.7	0.20
hsCRP (mg/L)	2.9 ± 2.9	2.2 ± 1.9	0.29

**Table 3:** Vitamin D-binding protein (DBP) baseline correlations in thirty patients with inflammatory skin disease. BMI (body mass index), hsCRP (high-sensitivity C-reactive protein), iPTH (intact parathyroid hormone), VAS (Visual Analogue Scale), PASI (Psoriasis Area and Severity Index), SCORAD (SCORing Atopic Dermatitis). *p*-values (correlation coefficient) are presented (Spearman’s correlation test).

Variable	DBP		
	All Patients (*n* = 30)	Psoriasis Patients (*n* = 20)	AD Patients (*n* = 10)
Age	0.62 (−0.09)	0.20 (0.30)	0.71 (−0.14)
BMI	0.62 (−0.09)	0.85 (−0.05)	0.95 (0.03)
hsCRP	0.25 (0.22)	0.96 (−0.01)	0.054 (0.64)
Total 25(OH)D	0.031 (0.39) *	0.21 (0.29)	0.080 (0.59)
Free 25(OH)D	0.045 (0.37) *	0.23 (0.28)	0.53 (0.23)
Percentage of free 25(OH)D	0.19 (−0.25)	0.34 (−0.23)	0.28 (−0.38)
1,25(OH)_2_D	0.0001 (0.65) ***	0.006 (0.59) **	0.30 (0.36)
iPTH	0.073 (−0.33)	0.63 (0.11)	0.028 (−0.71) *
VAS	0.11 (0.29)	0.11 (0.36)	0.58 (0.20)
PASI	N/A	0.92 (−0.02)	N/A
Objective SCORAD	N/A	N/A	0.84 (−0.07)

* *p* < 0.05, ** *p* < 0.01, *** *p* < 0.001.

## Data Availability

The data that support the findings of this study are available from the corresponding author upon reasonable request.
